# The translocator protein antagonist ONO-2952 attenuates neuroinflammation and neuronal apoptosis in an epilepsy rat model

**DOI:** 10.3389/fneur.2026.1924561

**Published:** 2026-08-24

**Authors:** Xiaojing Cheng, Hongli Liu, Bianbian Liang, Xiaodong Zhang

**Affiliations:** 1 Department of Neurology, Taiyuan Central Hospital of Shanxi Medical University, Taiyuan, China; 2 Changzhi Medical College, Changzhi, China; 3 Department of Neurology, The Second Hospital of Shanxi Medical University, Taiyuan, China

**Keywords:** epilepsy, neuroinflammation, ONO-2952, status epilepticus rat model, TSPO

## Abstract

Epilepsy is a globally prevalent chronic neurological disorder, and translocator protein 18 kDa (TSPO)-mediated neuroinflammation plays a critical role in epilepsy pathogenesis. This study aimed to explore the effect of the translocator protein 18 kDa antagonist ONO-2952 on neuroinflammation and cognitive function in epilepsy by establishing a mature epileptic rat model, which was divided into control group, untreated status epilepticus group and ONO-2952 intervention status epilepticus group; cognitive and behavioral tests were performed to observe the behavioral changes of rats, immunofluorescence staining was used to detect neuronal apoptosis, flow cytometry was adopted to analyze immune cell infiltration, and Western blotting was applied to measure the expression of proinflammatory cytokines. The results showed that ONO-2952 could down-regulate the expression of translocator protein 18 kDa, reduce the level of proinflammatory cytokines and peripheral immune cell infiltration, thereby inhibiting neuroinflammation in epileptic rats, and it could also relieve hippocampal neuronal apoptosis and effectively improve the cognitive dysfunction of model rats. This study confirms that ONO-2952 can regulate the expression of translocator protein 18 kDa, inhibit neuroinflammation and neuronal apoptosis, and improve cognitive function in epileptic rats, and further research is required to fully elucidate its specific biological effects.

## 1 Introduction

Epilepsy represents a prevalent neurological disorder, affecting an estimated 50 million individuals globally (1). Many antiepileptic drugs are currently used to manage seizures, but these drugs only relieve symptoms and cannot effectively stop disease progression (2, 3).

Epilepsy can be triggered by a single seizure, brain trauma, infection, or stroke; yet, only a small number of patients develop chronic epilepsy characterized by recurrent spontaneous seizures. This outcome relies on progressive pathological changes in the brain following the initial insult, including abnormal neuronal excitability, neural circuit remodeling, blood–brain barrier breakdown, inflammatory cell infiltration, gliosis, and extracellular matrix remodeling. These pathological alterations together form the basis of epileptogenesis and determine the transition from acute brain injury to chronic epilepsy. Therefore, exploring the molecular mechanisms underlying the pathological evolution of the brain after an initial brain injury or the first seizure and screening for key targets to halt this process represent the core scientific issues in epilepsy prevention and treatment.

An increasing number of recent studies have shown that neuroinflammation plays an important role in epilepsy (4, 5). Seizures can induce neuroinflammation, and repeated seizures maintain this inflammatory response. Such neuroinflammatory processes lead to neuronal damage, primarily by activating astrocytes and microglia and promoting the release of proinflammatory factors and other related molecules (6–8). For patients with drug-resistant epilepsy, targeting the signaling pathways involved in neuroinflammation may be an effective way to manage the disease (9).

Translocator protein 18 kDa (TSPO) is a mitochondrial protein that is present in the microglia of the central nervous system (CNS) (10, 11). It functions primarily to facilitate cholesterol transport across cell membranes, a process essential for neurosteroid synthesis. TSPO also plays a key role in regulating mitochondrial activity and apoptosis (12). In the normal brain, microglia express TSPO at a low basal level, but its expression increases sharply when microglia are activated under pathological conditions. In epilepsy, TSPO interacts with the voltage-dependent anion channel (VDAC) and the adenine nucleotide translocator (ANT) to form the mitochondrial permeability transition pore (mPTP) (13, 14); excessive mPTP opening leads to mitochondrial dysfunction and the release of pro-apoptotic factors such as cytochrome c and apoptosis-inducing factor (AIF), which activate apoptotic cascades (15). TSPO also promotes reactive oxygen species (ROS) generation, which amplifies mitochondrial damage (16, 17). Meanwhile, recurrent seizures trigger glial inflammation and activate the nuclear factor kappa-B (NF-κB) signaling pathway, which drives sustained TSPO overexpression (18). Upregulated TSPO in turn amplifies NF-κB-mediated inflammatory signals and activates the NLRP3 inflammasome, driving the production and release of key pro-inflammatory cytokines (19), which contribute to neuronal hyperexcitability, blood–brain barrier disruption, and neuronal apoptosis, thus forming a vicious feedforward loop that perpetuates neuroinflammation (20). Given its broad distribution in the CNS and elevated expression in neuroinflammatory states, targeting TSPO may serve as an effective approach to alleviate neuroinflammation and reduce neuronal damage (21).

ONO-2952 is a high-affinity ligand of TSPO (22). Previous studies have indicated that it promotes neural survival and decreases microglial activation after CNS injury (23, 24). However, no research has explored the effects of ONO-2952 on brain injury and inflammation in epilepsy. In this study, we demonstrated that ONO-2952 exerted anti-inflammatory effects, significantly reduced cytokine production, and improved neurological function in an epileptic rat model.

## 2 Materials and methods

### 2.1 Animals

Ethical clearance for all *in vivo* animal experiments was obtained from the Animal Ethics Committee of Zhonghong Boyuan Biotechnology Co., Ltd. All procedures followed the Laboratory Animal Care and Use Guidelines established in China. Male Sprague–Dawley rats, aged 6–8 weeks and weighing 250–300 g, were obtained from SPF (Beijing) Biotechnology Co., Ltd. The animals were kept under regulated housing conditions. Lithium–pilocarpine-induced epilepsy model and treatment. We used the lithium–pilocarpine method to induce status epilepticus (SE), as previously described (25) First, we administered lithium chloride (127 mg/kg) via intraperitoneal injection. Twenty-four hours later, methylscopolamine bromide (1 mg/kg) was administered to suppress peripheral cholinergic effects. Pilocarpine (30 mg/kg) was injected 30 min thereafter to induce seizures, which were scored according to the Racine scale. SE was confirmed when the animals exhibited stage IV–V convulsions that persisted for more than 30 min or failed to resume normal behavior during interictal intervals. Seizure activity was halted 60 min after onset with diazepam (10 mg/kg). After termination of SE, all rats were housed in a warm and dry environment to maintain body temperature and facilitate recovery. To prevent malnutrition and weight loss during the post-SE period, soft and palatable food supplements were provided to encourage feeding, and 5% glucose was administered intraperitoneally daily. Body weight was recorded daily during the first week after SE to monitor recovery.

ONO-2952 (MedChemExpress) was freshly prepared in 0.2 mg/mL phosphate-buffered saline (PBS). The rats were randomly allocated to three distinct experimental groups, with three animals in each group: (1) Control: received 0.9% saline (1 mL/kg) i.p. at time points matching SE induction; (2) SE: subjected to SE and treated daily by oral gavage with PBS (1 mL/kg); and (3) SE + ONO-2952: subjected to SE and administered ONO-2952 (1 mg/kg, p.o.) once daily, beginning 2 h before pilocarpine injection and continuing until euthanasia. All gavage doses were delivered at the same time each day (total of 3 administrations for rats euthanized at day 3 post-SE, and 7 administrations for those euthanized at day 7 post-SE).

### 2.2 Morris water maze assessment

The spatial learning and memory capacities of the experimental rats were evaluated via the Morris water maze test. The training began on day 3 post-SE and continued for four consecutive days (days 3–6), with four trials per day. On day 7 post-SE, a probe trial was conducted without the platform, and the animals were subsequently euthanized. In brief, a circular water pool with a diameter of 150 cm and a height of 70 cm was filled with opaque water, the temperature of which was maintained at 23 ± 1 °C. A submerged escape platform 10 cm in diameter was positioned 1.5 cm beneath the water surface in a fixed quadrant of the pool. Over four consecutive days, the rats completed four trials per day with 5-min intertrial intervals. Starting positions were counterbalanced across the north, east, south, and west quadrants daily, while the platform remained fixed. Animals unable to locate the platform within 90 s were guided to it and allowed to remain on it for 30 s. On Day 7, a probe trial was conducted without the platform. The time spent in the target quadrant and the number of crossings over the exact platform position were recorded as primary outcome measures.

### 2.3 Western blot analysis

Hippocampal samples were collected 72 h after SE initiation. Following the homogenization and centrifugation of the samples, the resulting supernatants were harvested, and protein concentrations were measured using the BCA method (Elabscience, Cat. No. E-BC-K318-M). Equal amounts of protein (30 μg) were separated by SDS–PAGE and transferred onto PVDF membranes (Millipore) via semidry blotting. The membranes were blocked overnight at 4 °C in PBS-T (0.1% Tween-20) supplemented with 10% nonfat milk and then probed with primary antibodies against TSPO, IL-1β, IL-6, and TNF-*α* (all 1:1,000; Proteintech or Abcam), with β-actin (15,000) used as a control. After three 10-min washes in PBS-T, the membranes were incubated with HRP-conjugated secondary antibodies (15,000, ZSGB-BIO) for 2 h at room temperature, followed by additional washes. An ECL reagent (Thermo Fisher Scientific, Cat. No. RJ239676) was used for chemiluminescence detection, and densitometry analysis was performed using ImageJ v1.48.

### 2.4 Immunofluorescence and TUNEL staining

Brain tissues for immunofluorescence and TUNEL staining were collected at day 7 post-SE, immediately after the Morris water maze probe trial. The tissues were fixed in a 4% paraformaldehyde solution were embedded in paraffin and cut into 5-μm-thick sections. After dewaxing, rehydration, and antigen retrieval, the slices were blocked for 1 h at room temperature using 5% goat serum in PBS. Next, they were treated overnight at 4 °C with a rabbit anti-NeuN primary antibody (1:200; Proteintech, Cat. No. 26975-1-AP), followed by treatment with fluorophore-conjugated goat anti-rabbit IgG secondary antibodies (Alexa Fluor 488 or CY3) for 1 h in the dark (ZSGB-BIO or Servicebio; dilution 1:100–1:1,500). Nuclei were stained with DAPI. To assess apoptosis, TUNEL staining was carried out using a red-fluorescence kit (Beyotime, Cat. No. C1090) following the manufacturer’s instructions. All images were captured on an Olympus BX53 fluorescence microscope. Quantification was performed in one or two randomly selected fields within the hippocampal CA1 region per section, with two sections analyzed per animal.

### 2.5 Flow cytometric analysis of brain-infiltrating leukocytes

On Day 3 post-SE, the brains were harvested and mechanically dissociated through a 40-μm cell strainer (BD Biosciences) in PBS. Single-cell suspensions were layered onto a discontinuous Percoll gradient (30% over 70%) and centrifuged at 2,000 rpm for 30 min. Mononuclear cells localized at the gradient interface were harvested, rinsed, and resuspended in FACS buffer (PBS + 1% BSA). Cells (1 × 10^6^ in 100 μL) were stained with the following fluorochrome-conjugated antibodies: anti-CD3-FITC (BioLegend, 201403), anti-CD4-PE/Cy7 (201515), anti-CD8-APC (200610), anti-CD45-PerCP/Cy5.5 (202220), anti-CD19-PE (Bioss, bs-0079R-PE), anti-CD11b-APC/Cy7 (Abcam, ab79096), anti-Ly6G-APC (Invitrogen, 17–9,668-82), and anti-CD68-FITC (Invitrogen, MA5-28262). Isotype controls were included for gating accuracy. Data were acquired on a NovoCyte 2060R flow cytometer (Acea Biosciences) and analyzed using FlowJo v10.

### 2.6 Statistical analysis

The data are expressed as the mean ± standard error of the mean (SEM). Each biological replicate corresponds to an individual rat, with *n* = 3 rats per group. The data were analyzed by one-way ANOVA followed by Tukey’s *post-hoc* test. Statistical significance was set at *p* < 0.05 (two-tailed), and all analyses were performed with GraphPad Prism 10.4.

## 3 Results

### 3.1 Assessment of status epilepticus severity

Rats in the control group exhibited no seizure behaviors throughout the observation period. In the SE and SE + ONO-2952 groups, the mean latency to reach stage IV–V seizures was 38.89 ± 2.62 min and 39.78 ± 2.68 min, respectively, with no statistically significant difference between the two groups (*p* > 0.05). The proportion of rats that progressed to the most severe stage V seizures was identical in both groups (55%, i.e., 5 out of 9 rats in each group). All rats included in the analysis reached stage IV–V seizures, confirming that the SE model met the experimental requirements.

### 3.2 TSPO is upregulated in epileptic rat brains, and ONO-2952 reverses this upregulation

**Figure 1:**
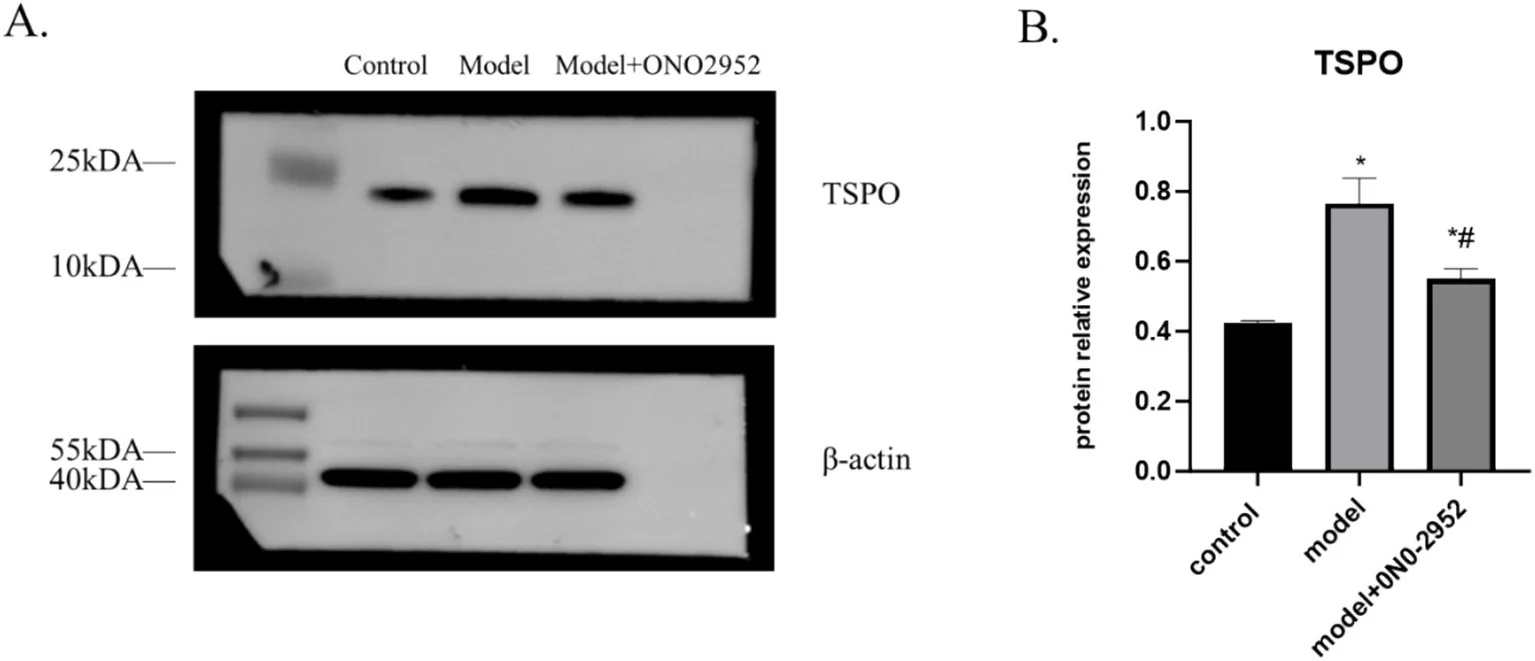
ONO-2952 downregulates TSPO expression in the brains of epileptic rats. **(A)** Western blotting showing decreased TSPO expression in epileptic rats following ONO-2952 treatment. **(B)** Quantification of TSPO protein levels in brain tissues across all groups is shown (*p < 0.05 vs. control; #*p* < 0.05 vs. SE).

To assess the expression level of TSPO after epilepsy induction and to study the effect of ONO-2952, we performed Western blot analysis on brain homogenates from the three experimental groups. Total protein was extracted and quantified using the BCA assay. As shown in Figure 1, TSPO expression was significantly higher in the SE model group than in the control group (*p < 0.05), confirming that seizure activity induces TSPO overexpression. In contrast, TSPO expression was markedly lower in ONO-2952-treated rats than in model rats (#*p* < 0.05), demonstrating that ONO-2952 effectively reverses seizure-driven TSPO upregulation.

### 3.3 ONO-2952 ameliorates cognitive deficits following SE

**Figure 2:**
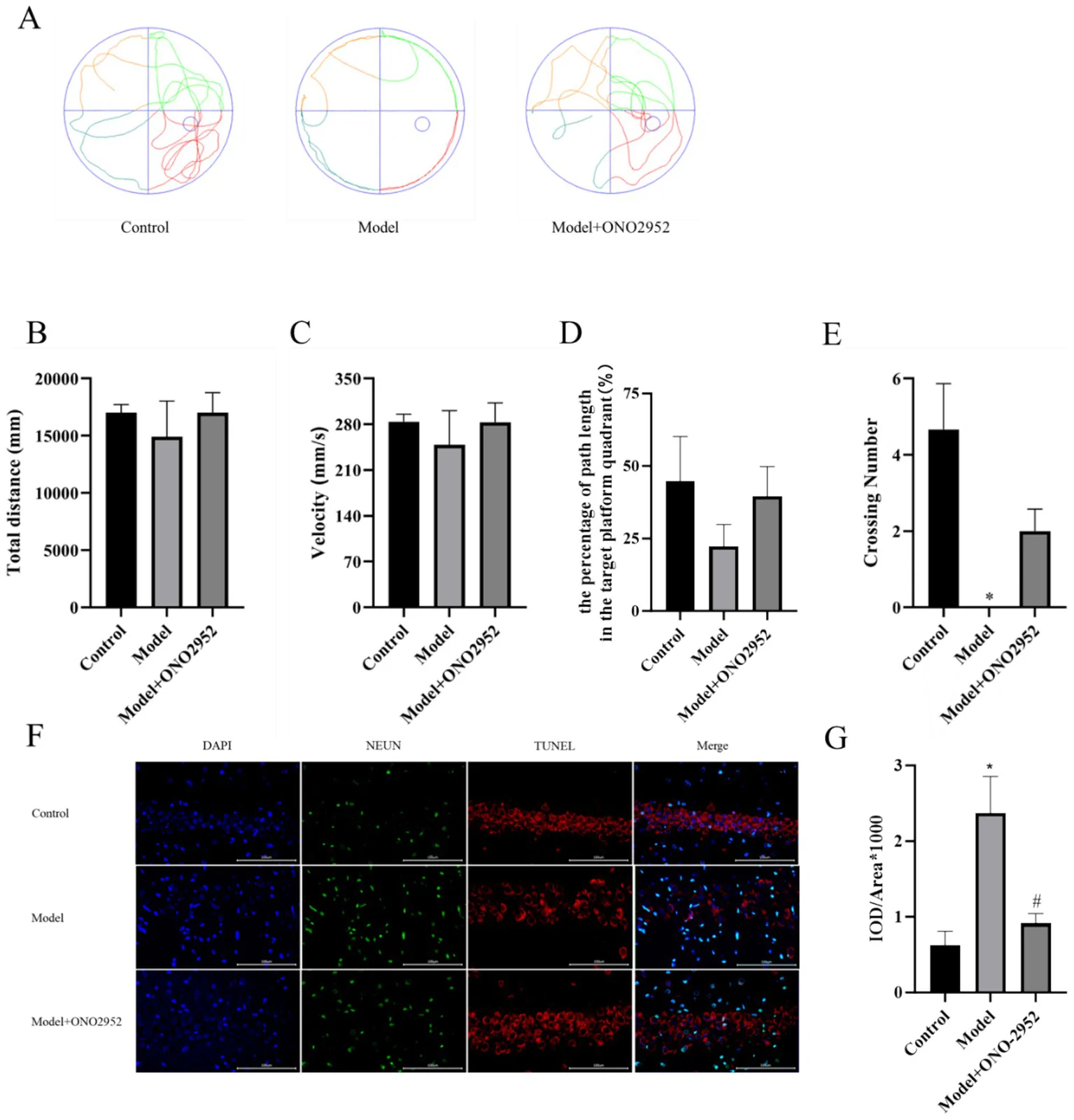
ONO-2952 ameliorates epilepsy-associated cognitive impairment and neuronal apoptosis. **(A)** Typical swim routes recorded for each group of rats in the Morris water maze test. **(B,C)** No significant differences were observed in total travel distance or mean swimming speed across groups during the testing phase. **(D)** Assessment of the proportion of path length spent in the target quadrant revealed no statistically significant difference; however, this was appeared lower in the epilepsy model group than in the control group, whereas the ONO-2952-treated group tended to recover. **(E)** The SE model group exhibited far fewer crossings over the original platform position (*p* < 0.05), and drug treatment effectively ameliorated this impairment, although the crossing number did not reach the level of the control group. **(F,G)** Double immunofluorescence staining of the hippocampus in rats from each group; scale bar = 100 μm. These results indicate that ONO-2952 can ameliorate neuronal apoptosis in epileptic rats. *p < 0.05 vs. control; #*p* < 0.05 vs. SE.

The Morris water maze test was used to assess spatial learning and memory abilities. Representative swim paths are presented in Figure 2A. To exclude potential confounding effects of motor dysfunction, total swim distance and average velocity were analyzed during the acquisition trials. No significant differences were observed across the control, SE, and ONO-2952–treated groups (Figures 2B,C) (*p* > 0.05), indicating intact locomotor function across all cohorts. During the probe trial, compared with the control group, the SE group exhibited a notable decrease in both the frequency of platform crossings and the percentage of path length traversed in the target quadrant (Figures 2D,E) (*p* < 0.05), reflecting impaired spatial memory retention. Compared with the SE group, the ONO-2952 treatment partially but significantly restored these parameters: the path length distribution improved (Figure 2D), and the number of platform crossings increased, although it did not fully return to control levels (Figure 2E).

Given the link between hippocampal neuronal loss and cognitive decline, we next assessed apoptosis in the CA1 region using double-labeling immunofluorescence for NeuN and TUNEL (Figure 2F). Compared with the control group, the SE group presented a marked increase in the number of TUNEL^+^/NeuN^+^ cells (*p < 0.05). ONO-2952 administration significantly attenuated neuronal apoptosis (#*p* < 0.05; Figure 2G). These data suggest that the cognitive benefits of ONO-2952 are associated with its neuroprotective effect against seizure-induced hippocampal cell death.

### 3.4 ONO-2952 reduces the expression of inflammatory factors in the brain tissues of epileptic rats

**Figure 3:**
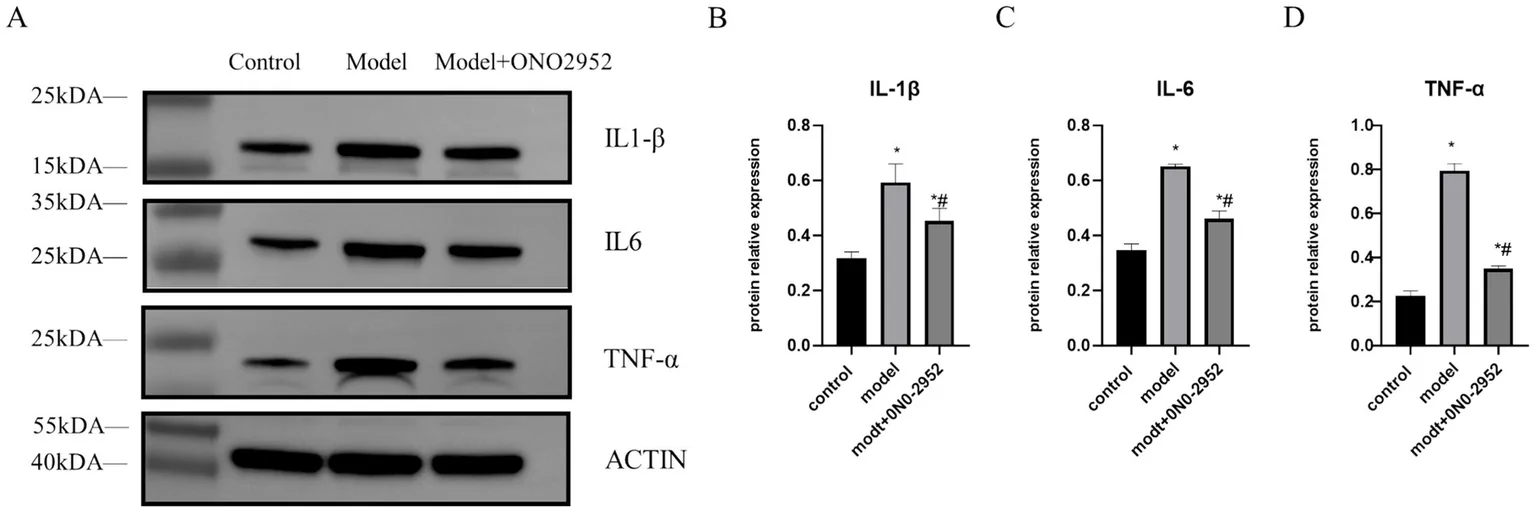
In epileptic rats, ONO-2952 reduces the levels of inflammatory mediators in brain tissue. **(A)** Representative Western blot bands showing the levels of IL-1β, IL-6, and TNF-*α* in brain tissue across groups: ONO-2952 administration markedly suppressed the upregulation of all three cytokines compared with those in the epilepsy model group. **(B–D)** Densitometric quantification of IL-1β, IL-6, and TNF-α levels. The data are expressed as the mean ± SEM (*p < 0.05 vs. control; #*p* < 0.05 vs. SE).

To explore how ONO-2952 modulates neuroinflammation in epileptic rats, we measured the levels of key inflammatory factors (IL-6, IL-1β, and TNF-*α*) in brain tissue. Compared with the control group, the epilepsy model group exhibited markedly elevated expression of IL-6, IL-1β, and TNF-α (Figure 3A). Notably, ONO-2952 treatment effectively decreased the levels of all these markers, indicating attenuated neuroinflammation. All the changes were statistically significant (*p < 0.05 and #*p* < 0.05).

### 3.5 ONO-2952 limits peripheral immune cell infiltration into the cerebral parenchyma

**Figure 4:**
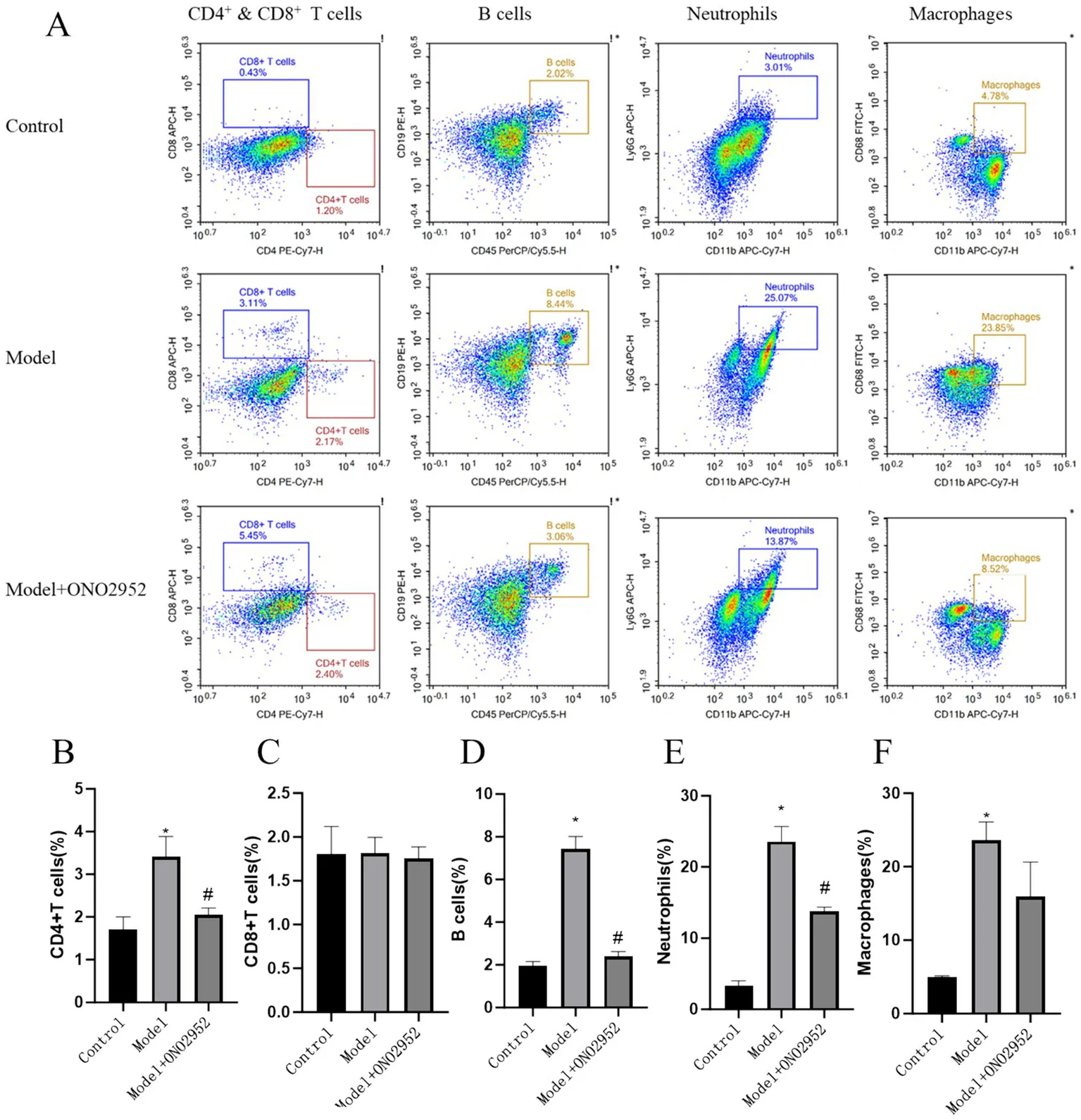
ONO-2952 reduces leukocyte infiltration in the brain after epilepsy. 3 days post-epilepsy induction, brain tissues were collected for single-cell isolation and subsequent flow cytometry. **(A)** The gating approach for distinguishing CD4^+^ T cells, CD8^+^ T cells, B cells, neutrophils, and macrophages is illustrated. **(B–F)** Proportions of the indicated leukocyte populations per experimental group (*p < 0.05 vs. control; #*p* < 0.05 vs. SE).

Emerging data suggest that peripheral immune cell infiltration is a critical contributor to postictal neuroinflammation. We therefore examined leukocyte recruitment into the brain 72 h after status epilepticus induction using flow cytometry. Compared with the control group, the epilepsy model group exhibited substantial increases in the numbers of brain-infiltrating CD4^+^ T cells (CD45hiCD3^+^CD4^+^), B cells (CD45hiCD19^+^), neutrophils (CD11b^+^Ly6G^+^), and macrophages (CD11b^+^CD68^+^) (Figures 4B–D) (*p < 0.05). ONO-2952 treatment markedly reduced the proportions of these subsets (#*p* < 0.05), suggesting the inhibition of peripheral immune cell trafficking across the blood–brain barrier. The proportion of cytotoxic CD8^+^ T cells remained unchanged following ONO-2952 administration (Figure 4C).

## 4 Discussion

Epilepsy is among the most prevalent chronic neurological disorders and neuroinflammation has been increasingly recognized as a critical contributor to its pathogenesis. However, effective therapeutic strategies targeting inflammatory pathways remain limited. In the present study, we investigated whether ONO-2952, a selective TSPO antagonist, could alleviate neuroinflammation and improve cognitive function in a lithium-pilocarpine-induced rat model of epilepsy. We recorded core acute seizure parameters, and no statistically significant differences were observed between the SE and SE + ONO-2952 groups in the latency to stage IV–V seizures or the proportion of rats developing stage V seizures. Given the limited sample size and the restricted seizure-related indicators in this study, we cannot draw definitive conclusions regarding whether ONO-2952 possesses direct anticonvulsant activity.

TSPO is a well-established marker of neuroinflammation, with its expression significantly upregulated in activated microglia under pathological conditions (17). In our study, we observed a marked increase in TSPO expression in the brain tissues of epileptic rats, consistent with previous findings in epilepsy and other neuroinflammatory disorders. Notably, treatment with ONO-2952 effectively reversed this upregulation, confirming its target engagement in the epileptic brain. Beyond its role as a biomarker, TSPO has been shown to regulate mitochondrial function and apoptosis through its interaction with VDAC and ANT to form the mPTP (13). Our TUNEL staining results further demonstrated that ONO-2952 treatment significantly reduced the number of apoptotic neurons in the hippocampal CA1 region, suggesting that TSPO antagonism may confer neuroprotection by preserving mitochondrial integrity and inhibiting downstream apoptotic cascades. Additionally, the reduced expression of IL-1β, IL-6, and TNF-*α* in ONO-2952-treated rats indicated a suppression of the inflammatory cascade, which was further supported by the diminished infiltration of peripheral immune cells into the brain parenchyma. Collectively, these findings suggest that ONO-2952 exerts neuroprotective and anti-inflammatory effects in the epileptic brain, likely through the modulation of TSPO-mediated mitochondrial and inflammatory pathways (26).

Since most patients still experience cognitive impairments after epileptic seizures are controlled, we tested the spatial memory of the rats following epilepsy induction using the Morris water maze. Compared with the control rats, the SE rats crossed the platform location significantly fewer times, confirming the cognitive impairment associated with epilepsy. In contrast, ONO-2952 treatment restored cognitive performance. Histological evidence revealed that ONO-2952 decreases the apoptosis of hippocampal cells, and the improvement in cognition in the rats is consistent with this effect.

Microglial activation is a hallmark of neuroinflammation, and activated microglia contribute to neurotoxicity through the secretion of various proinflammatory mediators. In the present study, we focused on three of the most established and mechanistically relevant pro-inflammatory cytokines—IL-1β, IL-6, and TNF-*α*—which are consistently reported to be elevated in epilepsy and are widely used as key readouts in neuroinflammatory studies. Our results demonstrated that ONO-2952 significantly suppressed the expression of these representative cytokines, further supporting its anti-inflammatory effects in the epileptic brain. This, together with the observed reduction in peripheral immune cell infiltration, aligns with the emerging concept that the brain is not immunologically isolated but dynamically interacts with the systemic immune system.

Although resident glia contribute to neuroinflammation in epilepsy, glial activation alone might not be sufficient (27–29). Infiltrating immune cells are involved in the pathology: neutrophils invade acutely after SE, monocyte counts correlate with seizure frequency, and CD8^+^ T cells invade epileptogenic foci, where their accumulation in the hippocampal CA1 region is associated with neuronal loss (30). We show that ONO-2952 decreases such infiltration, suggesting an additional explanation for its neuroprotective efficacy. Beyond its role in neuroinflammation and immune modulation, TSPO has also been implicated in the regulation of redox balance in previous studies, although the underlying mechanisms remain incompletely understood (26, 31).

Several limitations of the current study should be acknowledged. First, all biological detection and behavioral assessments were performed during the acute phase after pilocarpine-induced status epilepticus, and long-term monitoring of chronic spontaneous seizures was not carried out. Therefore, only acute pathological alterations triggered by early brain injury were characterized, and we could not observe long-term persistent changes in neuroinflammation and cognitive function during chronic epileptogenesis. Second, ONO-2952 was administered prophylactically 2 h before SE induction in this study. This pretreatment strategy was adopted to fully clarify the causal association between TSPO upregulation and the initiation of neuroinflammatory cascades. However, this administration timing fails to mimic clinical scenarios where interventions are initiated after seizure onset, which limits the direct translational reference value of the present dosing regimen. Third, the present findings were obtained solely in the lithium-pilocarpine model, and further validation in other epileptic models is required.

## 5 Conclusion

In summary, ONO-2952 attenuates neuroinflammation and ameliorates cognitive impairment in epileptic rats. The underlying mechanisms include inhibiting cytokine release, limiting the recruitment of peripheral immune cells, and protecting hippocampal neurons. These results suggest that TSPO represents a promising therapeutic target for epilepsy and validate ONO-2952 as a potential candidate for adjunctive treatment.

## Data Availability

The raw data supporting the conclusions of this article will be made available by the authors, without undue reservation.
